# Royal jelly with ellagic acid inhibits the glycolytic pathway and induces apoptosis through multiple pathways in colorectal cancer

**DOI:** 10.55730/1300-0152.2770

**Published:** 2025-09-08

**Authors:** Tuğba KUL KÖPRÜLÜ

**Affiliations:** 1 Experimental Medicine Application and Research Center, University of Health Sciences, İstanbul, Turkiye; 2 Department of Molecular Medicine, Hamidiye Institute of Health Sciences, University of Health Sciences, İstanbul, Turkiye

**Keywords:** xCELLigence, Seahorse XFe24, apoptosis, transcriptome, colorectal cancer

## Abstract

**Background:**

Developing novel chemotherapeutics with high anticancer efficacy and low toxicity remains a critical challenge in oncology. Natural products have shown promise due to their multitargeted activity and favorable safety profiles. The present study investigates the combined anticancer effects of royal jelly (RJ) and ellagic acid (EA), two potent antioxidants of animal and plant origin.

**Materials and methods:**

Royal jelly (RJ) and ellagic acid (EA) were applied to HT29 (ATCC HTB-38, Human colorectal adenocarcinoma), HCT116 (ATCC CCL-247, Human colorectal carcinoma) and BEAS-2B (ATCC CRL-3588, human bronchial epithelium) cell lines, and their antiproliferative effects were evaluated using a xCELLigence Real-Time Cell Analyzer (RTCA MP). The effect of the combination of RJ and EA on the glycolytic pathway was determined using a Seahorse XFe24 Analyzer, and the apoptotic process was evaluated by DNA laddering and the expression of the *Bcl-2* and *Bax* genes in the apoptotic pathway through real-time quantitative PCR (RT-qPCR). The transcriptome profiling of the combination of RJ and EA on colorectal cancer cells was performed by Total RNA Sequencing analysis.

**Results:**

RJ with EA, when used in combination, significantly reduced the extracellular acidification rate (ECAR), effectively inhibiting aerobic glycolysis, especially in HCT116, and induced apoptosis in HCT116 and HT29 cells by increasing the *Bax*/*Bcl-2* ratio compared to cases treated with EA or RJ alone (p < 0.05). GSEA analyses revealed that the treatment of both cell lines increased the expression of apoptosis and p53 pathway-related genes while suppressing the genes associated with the E2F target, G2M checkpoint, oxidative phosphorylation, and MYC target mechanism, indicating a directly proportional relationship with the antiproliferative effect on cancer cells and increased apoptosis.

**Conclusion:**

RJ with EA used in combination demonstrates potent anticancer effects in colorectal cancer by suppressing glycolysis and activating apoptosis, with apparent therapeutic potential as a novel cancer treatment strategy.

## 1. Introduction

Colorectal cancer (CRC) is the third most common type of cancer in the world after lung and breast cancer, and the number of CRC cases is projected to reach 3.2 million by 2040 (Xi and Xu, 2021; Morgan et al., 2023). Obesity, fat-rich diet, sedentary lifestyle and smoking are significant risk factors for colorectal cancer (Xi and Xu, 2021). Treatment options for CRC include surgery, radiotherapy, chemotherapy, and targeted therapies, with surgical intervention preferred in early-stage patients, and chemotherapy in cases with advanced-stage and metastatic colon cancer (Chakrabarti et al., 2020; Xi and Xu, 2021; Leowattana et al., 2023). The therapeutic effects of chemotherapy are accompanied by numerous side effects, and tumors can rapidly develop resistance to chemotherapeutic agents (Lin et al., 2020; İslam et al., 2022; Wang et al., 2022). There is thus a need to identify new chemotherapy agents with treatment potential but with fewer toxic effects (İslam et al., 2022; Tanaka et al., 2023). Since more than one signaling pathway is active in cancer development, agents that can suppress multiple pathways should be evaluated. Natural products are attracting significant interest in the search for new chemotherapeutic drugs for the treatment of cancer due to their various health benefits, as well as their low toxicity and side effects (Tanaka et al., 2023; Thompson and Lutsiv, 2023). Approximately 50% of all cancer drugs originate from natural products (Calixto, 2019; Asma et al., 2022). Phytochemicals, bioactive compounds produced by plants for self-protection, can help prevent cancer by targeting multiple pathways affecting physiological processes. In particular, polyphenols—secondary metabolites commonly found in plants—are responsible for the high antioxidant properties of natural plants (Kumar et al., 2016; Tanaka et al., 2023). Ellagic acid (EA) is a polyphenol with extremely strong antioxidant properties that is found in fruits such as pomegranate, strawberry, raspberry, hazelnut, and walnut (Čižmáriková et al., 2023; Lu et al., 2023; Maleki et al., 2023; Ni et al., 2023). The lipophilic properties of EA stem from the four aromatic rings in its structure, while the four hydroxyl groups and two lactone groups it contains creates a hydrophilic area within the molecule (Kaur et al., 2021; Deepika and Maurya, 2022). This structure allows EA to accept electrons from various substrates, contributing to its strong antioxidant profile (Ríos et al., 2018; Čižmáriková et al., 2023). That said, the low water solubility, permeability, and lipophilic character of EA limit the bioavailability of EA (Tanaka et al., 2023). EA possesses a wide range of beneficial properties, with antioxidant (Kumar et al., 2016; Ríos et al., 2018; Chen et al., 2019; Tošović and Bren, 2020), antiviral (Park et al., 2014; Evtyugin et al., 2020), antiinflammatory (Chen et al., 2019; Gil et al., 2021; Lu et al., 2023), antimutagenic (Zahin et al., 2014), antimicrobial (Savic et al., 2019), and antifibrotic effects (Mannino et al., 2023), and also protects the cell against lipid peroxidation and oxidative damage (Mansouri et al., 2020; Maleki et al., 2023; Naghibi et al., 2023). Furthermore, EA can bind and interact with such important macromolecules as proteins, enzymes, and DNA (Muthukumaran et al., 2017). Although there are publications in the literature reporting on the anticancer activity of EA (Ceci et al., 2018; Mohammadinejad et al., 2022), further studies are needed to expand the current knowledge (Li et al., 2018; Mohammadinejad et al., 2022; Lu et al., 2023) and contribute to the development of new ideas for its use. Another substance of interest that has emerged in this field is royal jelly (RJ) – a honey bee secretion that is used to nourish their larvae and that is critical to queen bee development. This yellowish, gelatinous-viscous bee product, secreted from the hypopharyngeal and mandibular glands of worker honeybees, has high phenolic content. Larvae fed with RJ for only the first 3 days emerge as worker bees, while larvae fed with RJ for longer periods emerge as queen bees (Shakib Khoob et al., 2022; Alhosin, 2023). The nutritional effects of RJ on bees are remarkable. Worker bees fed with RJ for only 3 days remain infertile and have a lifespan of approximately 40 days, while fertile queen bees live for 5–6 years. RJ has a highly nutritious composition, with proteins, vitamins, minerals, and phenolic and flavonoid compounds in its structure. The high antioxidant capacity of RJ (Zhang et al., 2017) can contribute to the reduction of oxidative stress and the correction of mitochondrial dysfunctions (Baptista et al., 2023). Mitochondrial dysfunction, which has been linked to various diseases, including cancer, causes oxidative stress and cellular damage as a result of energy imbalance and the release of reactive oxygen species (ROS). Studies have reported the antioxidant potential of RJ to can enhance the effectiveness of chemotheraputics when used together with anticancer drugs. In a study conducted by Borawska et al., temozolomide, a chemotherapeutic used for the treatment of brain cancer, synergistically increased the cytotoxic effect on U87MG cells when used with RJ (Borawska et al., 2014). RJ has been shown to significantly decrease the nephrotoxic and hepatotoxic side effects of cisplatin, a common cancer treatment with numerous side effects (Moubarak et al., 2021; Salama et al., 2022). Furthermore, studies have shown that the adverse effects of cyclophosphamide—a common breast cancer chemotherapy—can be eliminated through the co-administration of RJ (Albalawi et al., 2021; Moubarak et al., 2021). In another study, the effectiveness of 5-Fluorouracil in human colon cancer HCT116 was noted to increase when used with RJ (Salama et al., 2022). High drug doses are administered for the treatment of cancer, and nonpharmacological adjuvants such as RJ can increase their effectiveness. It has been noted that response rates to cancer treatments are decreasing as a result of the increasing resistance to existing cancer therapeutics, necessitating the further investigation of new therapeutics and their synergistic effects (Mansourizadeh et al., 2020). The use of natural products containing polyphenols and flavonoids alongside chemotherapy treatments may represent a promising strategy for cancer treatment. The use of quercetin and resveratrol in combination has been studied in human pancreas and squamous-cell carcinomas, and has shown anticancer activity. Furthermore, curcumin combined with resveratrol increased the apoptotic effect in hepatocellular carcinoma (Kumar et al., 2016). Curcumin-loaded apoferritin nanoparticles in combination with quercetin induced apoptosis and increased antitumor activity against human breast cancer cells (Patra et al., 2021). In the present study, the potential anticancer activity and mechanisms of the combined use of EA and RJ, which have high antioxidant properties, on HCT116 and HT29 cells were investigated.

## 2. Materials and methods

### 2.1. Cell culture conditions

The BEAS-2B (human lung bronchial epithelial cell, ATCC CRL-3588), HCT116 (The human colorectal carcinoma, ATCC CCL-247) and HT29 (The human colorectal adenocarcinoma, ATCC HTB-38) cell lines for the present study were sourced from ATCC (Manassas, VA). The cells were routinely cultured in high glucose Dulbecco’s Modified Eagle Medium (DMEM) supplemented with heat-inactivated 10% FBS, 1% L-glutamine, and 2% penicillin-streptomycin at 37 °C in a humidified atmosphere of 5% CO_2_ until the cells reached 80% confluence. The cells were regularly tested for mycoplasma contamination. After a cell line reached approximately 80% confluency, Trypsin-EDTA (0.25%) was added to detach the cells from the surface. The cells were then stained with trypan blue and counted with a TC20 automated cell counter (BioRad, USA). RJ was obtained from Macahel (Artvin, Türkiye) and dissolved in PBS for 1 h at 4 °C for the preparation of a 1 g/mL stock solution. The mixture was then centrifuged at 12,000 × *g* for 10 min at 4 °C, and the collected supernatant was filtered and stored at 4 °C until required for the experiment. The EA (sourced from Sigma-Aldrich, Germany) was dissolved in DMSO (Sigma-Aldrich, Germany) at a concentration of 100 mM. After centrifugation, the supernatant was filtered through a 0.22 μm syringe filter and stored at −20 °C.

### 2.2. Monitoring real-time cell proliferation

The cell proliferation of the BEAS-2B, HCT116 and HT29 cells was performed in real time using the xCELLigence RTCA MP system (Agilent Technologies, USA), placed in a humidified incubator at 37 °C and 5% CO_2_. To measure background impedance, cell culture medium was added to each well of the E-plate. After measuring the background control and determining the optimal seeding density, BEAS-2B cells were seeded at a density of 1.5×10^4^ cells per well, HCT116 cells at a density of 4×10^4^ cells per well, and HT29 cells at a density of 1×10^4^ cells per well into 96-well E-plates, with a total volume of 200 μL per well. The E-plates were then incubated in the xCELLigence instrument for 24 h to allow the cells to attach to the surface. The cells were then treated with six different concentrations of RJ and EA in triplicate, ranging from 5–30 mg/mL and 10–60 μM, respectively. For each cell line, cells were left without RJ and EA treatment as the control group. Cell impedance was monitored using the xCELLigence system for at least 96 h. The xCELLigence system detects the electrical impedance resulting from the adherence of a eukaryotic monolayer to the bottom of the test plate, and any changes in electrical impedance are converted into a Cell Index (CI). An increase in electrical impedance leads to a higher cell index, and so as the number of adherent cells increases, so does the cell index. Impedance measurements were taken every 15 min and a time-dependent CI graph was generated by the device using RTCA Software Pro 2.3.4 (Agilent Technologies, 2020).

### 2.3. ECAR Measurement

Glycolysis is the process by which glucose is broken down in the cell to produce energy, and is a key metabolic pathway that cancer cells prefer even in the presence of oxygen, making it a modulator of tumor formation. During glycolysis, glucose is converted into lactate and protons are exported to the environment. The increase in protons outside the cell, as the extracellular acidification rate (ECAR), is an indicator of glycolysis. The ECAR (mpH/min) was measured in real time with a Seahorse XF Glycolysis Stress Test Kit using the Seahorse XFe24 Analyzer (Agilent Technologies) according to the manufacturer’s instructions. Briefly, HCT116 cells were seeded at 5×10^4^ cells per well and HT29 at 4×10^4^ cells per well in 24-well plates and incubated for 24 h and 42 h for HCT116 and HT29, respectively. The next day, their baseline measurements were determined and glucose (10mM), oligomycin (1 μM), and 2-deoxyglucose (2-DG, 50 mM) were injected sequentially using the Seahorse XFe24 Analyzer. The data were analyzed using Seahorse Wave Controller Software 2.4 (Agilent Technologies, 2020). This XF instrument control software contains predefined analysis templates and an interface to simplify metabolic analyses. Raw data from the Seahorse XFe24 Analyzer were first imported into the Wave Controller Software 2.4, plate layout and group assignments (e.g., control and treatment groups) were performed, and basic adjustments were made to mitigate the background noise. Wells with poor signal consistency or technical errors (air bubbles) were flagged and excluded from the analysis. The Oxygen Consumption Rate (OCR), reflecting mitochondrial respiration in pmol/min, and ECAR (Extracellular Acidification Rate), reflecting glycolytic activity in mpH/min, were then analyzed by the software as real-time kinetic OCR and ECAR plots over time.

### 2.4. DNA laddering assay

Apoptosis, or programmed cell death, is a process that is frequently suppressed in cancer cells and is characterized by certain morphological changes in the cell, such as chromatin condensation, the formation of apoptotic bodies, and nuclear shrinkage. Apoptotic DNA fragmentation is able to be observed to have a ladder pattern. DNA laddering, consisting of 180–200 bp resulting from DNA cleavage, can be visualized for the detection of cytogenetic damage and apoptosis. That analyzed fragments have a characteristic ladder-like pattern in agarose gel electrophoresis, which serves as an indicator of the apoptotic process. The intensity of the ladder is directly correlated with the apoptotic cell count. In contrast, a dense, diffuse smear observed on the gel is indicative of necrotic cell death. To observe apoptosis in the study, HCT116 and HT29 cells were seeded at 7.5×10^5^ in T25 cell culture flasks. The cells were then treated with 25 mg/mL RJ and 50 μM EA for 24 hours in HCT116, and 20 mg/mL RJ and 40 μM EA for 42 hours in HT29. The control group included untreated cells that were incubated only with a cell culture medium. An IC_50_ dose of the Paclitaxel (PAX) chemotherapeutic drug was used as a positive control. After incubation, adherent and non-adherent cells were collected using a cell scraper, and were centrifuged at 1500 rpm for 5 min to pellet the cells, and fixed in 70% ethanol at -20 °C for 48 h. The cell suspension was then centrifuged at 1200 rpm for 5 min and the resulting cell pellet was left to dry in the incubator at 37 °C. The cell pellet was then resuspended with a 0.2 M phosphate-citrate buffer at pH 7.8 and treated sequentially with RNase A (0.1 mg/mL) and Tween 20 at 37 °C for 30 min. The DNA was then electrophoresed in a 2% agarose gel containing ethidium bromide, and the DNA laddering in the gels was viewed using the Chemidoc MP Imaging System (BioRad, USA).

### 2.5 RT-qPCR for apoptosis-associated genes

The HCT116 cells were treated with 25 mg/mL RJ and 50 μM for 24 hours, and the HT29 cells with 20 mg/mL RJ and 40 μM EA for 42 h. Total RNA was extracted using a Total RNA MiniPrep Kit (BioBasic, Canada), and the total RNA concentration was determined using the Qubit RNA Broad Range Assay Kit (Invitrogen, USA) at Qubit 4.0 fluorometer. The RNA Integrity Number (RIN) was recorded using an RNA ScreenTape Assay and a Tapestation 4150 (Agilent Technologies, USA). Total RNA was converted into cDNA using the RT^2^ First Strand Kit (Qaigen, Germany). An RT-qPCR was performed in a Roche Light Cycler 480 using 2 ng/uL of cDNA for each sample, the RT^2^ SYBR Green Fast Master Mix (Qiagen, Germany), and the following primers from Integrated DNA Technologies (IDT, USA) (Table 1). MRNA expression was normalized through the expression of the GAPDH housekeeping gene, and data was acquired and statistics were produced using GeneGlobe Data Analysis Software (Qiagen, Germany). The relative expression levels of mRNA were calculated using the comparative cycle threshold 2^−ΔΔCt^ method.

### 2.6. RNA isolation, library preparation and RNA sequencing

Total RNA libraries were created to profile the total transcriptome of the HCT116 and HT29 cells affected by RJ and EA. First, the RNAs of the HCT116 cells treated with 25 mg/mL RJ and 50 μM EA for 24 h and the HT29 cells treated with 20 mg/mL RJ and 40 μM EA for 42 h were isolated using the RNA extraction kit following the manufacturer’s protocol (Biobasic, Canada). The obtained RNAs were then measured using Qubit 4.0 (Thermo Fisher Scientific, USA) fluorometer and TapeStation 4150 (Agilent, USA) and 700–1000 ng of RNA and RIN values between 6.0–10.0 were obtained. The isolated RNAs were used for the library preparation in concentrations of 100 ng. A QIAseq FastSelect RNA (Qiagen, Germany) kit was used following the manufacturer’s protocol. cDNAs were generated following rRNA removal. The samples were then indexed with 10 base-pair sequences, bound with magnetic beads, and washed with ethanol. At the end of the study, libraries of approximately 100 nM had been created. After pooling the libraries to a final concentration of 2 nM, the pool was sequenced with NovaSeq 6000 (Illumina, USA).

### 2.7. Functional enrichment analyses of DEGs

The Differentially Expressed Genes (DEGs) in the HT29 and HCT116 cells were determined separately using the QIAGEN CLC Genomics Workbench v. 25.0.2 (https://digitalinsights.qiagen.com). The gene set enrichment analyses (GSEA) of both DEGs were performed using R software (version 4.4.2) (FDR < 0.05 filtered data). Hallmark gene sets were used in the GSEA analyses to reveal the functions and regulatory relationships of specifically deregulated genes in more depth, and a significance threshold of P < 0.05 was set for the identification of significantly enriched gene sets. The STRING database was used for the independent gene ontology and PPI analyses for up-down genes using DEGs with |log2FC| > 1 and FDR < 0.05, and Cytoscape (v3.10.3) was used to perform and visualize MCODE in the PPI analysis using the following parameters: degree cutoff: 2, K-Core: 2, node score cutoff: 0.2, maximum depth: 100. The major clusters in the MCODE studies were subjected to KEGG pathway analyses, and a heatmap analysis of the top 20 up-down genes was also carried out. A Venn diagram tool was used to identify the genes common to both cell lines following treatment, and a KEGG pathway analysis was performed for the identified common genes using the STRING tool.

### 2.8. Statistical analysis

The results from three to five separate experiments were expressed as mean ± standard deviation (SD). All data collected from experiments were performed in triplicate and analyzed using the GraphPad Prism Version 10.2.3 program (GraphPad Software, Boston, Massachusetts USA). A two-way ANOVA was conducted, followed by Tukey’s multiple comparisons test for group-wide comparisons.

## 3. Results

### 3.1. Effects of EA, RJ, and their combination on cell proliferation in HCT116 and HT29 cells

**Figure 1:**
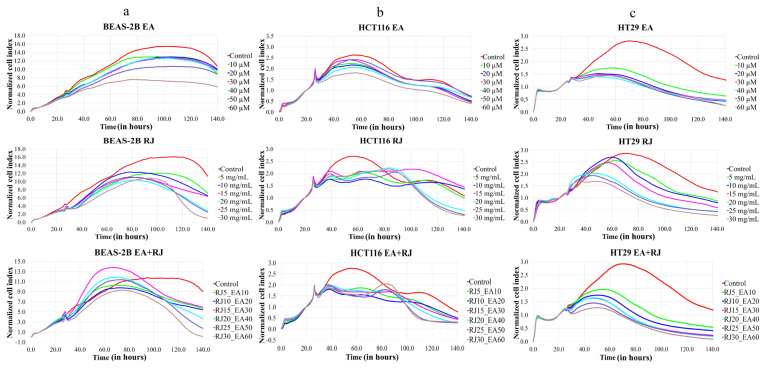
Evaluation of antiproliferative and cytotoxic effects using real-time cell analysis: determination of antiproliferative and cytotoxic effects of EA, RJ and EA-RJ combination in BEAS-2B (A) human cells, HCT116 (B) and HT29 (C) human cancer cells by real-time cell analysis: EA at doses of 10 μM, 20 μM, 30 μM, 40 μM, 50 μM and 60 μM and RJ at doses of 5 mg/mL, 10 mg/mL, 15 mg/mL, 20 mg/mL, 25 mg/mL and 30 mg/mL, applied to the cells alone and together.

Cell growth was monitored for up to 140 hours using the xCELLigence system to assess the antiproliferative effects of EA, RJ, and their use in combination, on HCT116 and HT29 cancer cells and healthy BEAS-2B cells. EA was applied to cells in concentrations of 10, 20, 30, 40, 50, and 60 μM, and RJ was applied in concentrations of 5, 10, 15, 20, 25, and 30 mg/mL. An analysis of the results revealed that EA, RJ, and their use in combination exerted greater antiproliferative effects on HCT116 and HT29 cells when applied separately, than on BEAS-2B cells, depending on the dose and time (Figure 1). The IC_50_ of the EA values were recorded as 45.8 μM in HCT116 cells and 25.7 μM in HT29 cells by xCELLigence RTCA MP. The concentration- and time-dependent antiproliferative activity of EA could be clearly observed in the BEAS-2B cells for approximately 96 h. It is interesting to note that although EA also exhibited dose-dependent antiproliferative activity in HCT116 and HT29 cells, the antiproliferative activity of EA was much faster in the HCT116 and HT29 cells than in the BEAS-2B cells. While prolonged antiproliferative activity was noted for RJ in BEAS-2B cells, depending on dose and time, its antiproliferative activity started much earlier in HCT116, and in HT29 in particular. Cotreatments with RJ (25 mg/mL for HCT116, 20 mg/mL for HT29) and EA (at IC_50_ doses) synergistically suppressed proliferation, and exhibited significantly enhanced antiproliferative activity when compared to treatments with the individual agents (p < 0.05). The fact that the EA-RJ combination showed faster and greater antiproliferative activity in HT29 cells than in HCT116 cells indicates that HT29 cells are more sensitive to the applied combination.

### 3.2. Assessment of EA and RJ and their use in combination on glycolytic function on cancer cells using the Seahorse XFe24 Analyzer

**Figure 2:**
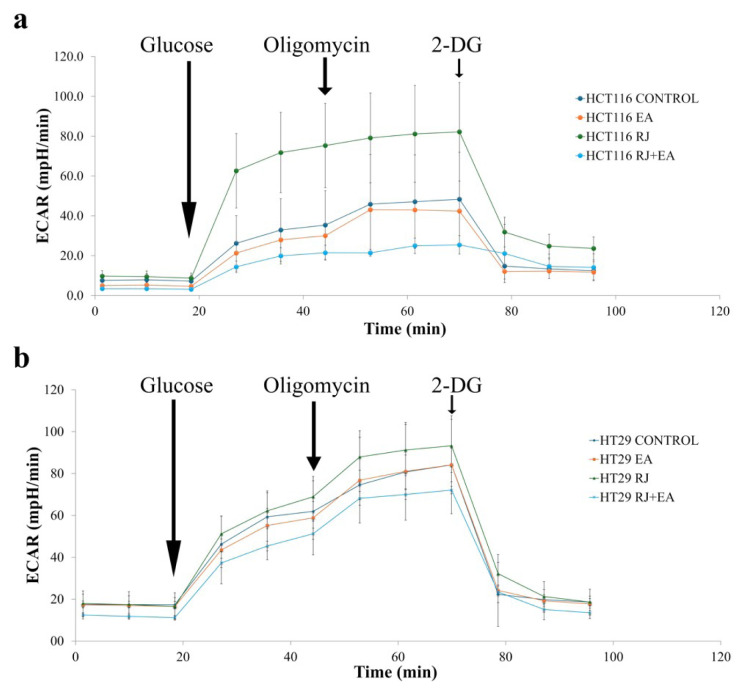
Representative results of ECAR of HCT116 (a) and HT29 (b) colorectal cancer cells treated with 50 μM EA (orange), 25 mg/mL RJ (green), 50 μM EA and 25 mg/mL RJ combination (light blue) for 24 h. Bars, mean ± SEM. p < 0.05.

Cancer cells are known to prefer the glycolytic pathway for the fulfillment of their rapid energy needs. The effect of the combined use of EA and RJ on glycolytic function (glycolysis, glycolytic capacity, and glycolytic reserve) in cancer cells was analyzed using the Seahorse XFe24 Analyzer. As can be seen in Figure 2, the addition of glucose to the medium led to an increase in ECAR in both cell lines. The findings show that EA used in combination with RJ suppressed glycolysis in HCT116 cells. 2-DG, a canonical glycolytic inhibitor, was used to validate the assay. The addition of oligomycin (1 μM) to the medium, which binds to the F0 subunit and cuts off the proton flow to inhibit ATP synthase, generally led to greater lactate production in both cell lines and revealed their maximum glycolytic capacity. The addition of oligomycin to the EA-RJ combination led to a greater reduction in ECAR than achieved with EA and RJ alone. The EA-RJ combination led to a considerably greater reduction in the ECAR rate in the HCT116 cell line.

### 3.3. Evaluation of apoptotic potential with DNA laddering and morphological changes

**Figure 3:**
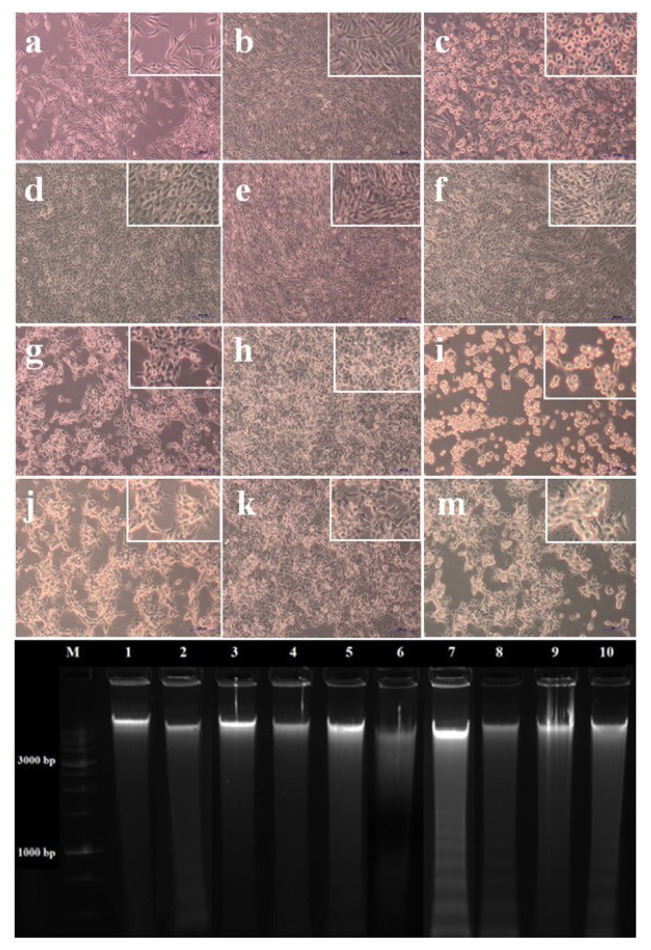
Observation of cell morphology under light microscope (up) and detection of apoptotic DNA Ladder in HT29 and HCT116 colorectal cancer cells (down). a: Untreated HT29 cells (Control, 0 h); b: Untreated HT29 cells (Control, 42 h); c: PAX+HT29 cells (42 h); d: RJ (20 mg/mL)+HT29 cells (42 h); e: EA (40 μM)+HT29 cells (42 h); f: RJ (20 mg/mL)+ EA (40 μM)+HT29 cells (42 h); g: Untreated HCT116 cells (Control, 0 h); h:Untreated HCT116 cells (Control, 24 h); i: PAX+HCT116 cells (24 h); j: RJ (25 mg/mL)+HCT116 cells (24 h); k: EA (50 μM)+HCT116 cells (24 h); m: RJ (25 mg/mL)+ EA (50 μM)+HCT116 cells (24 h). M: Marker (1 kb DNA Ladder); Lane 1: HT29 Control (Untreated cells, negative control); Lane 2: PAX-treated HT29 cells (positive control); Lane 3: RJ (20 mg/mL)-treated HT29 cells; Lane 4: EA (40 μM)-treated HT29 cells; Lane 5: RJ (20 mg/mL) and EA (40 μM) combination-treated HT29 cells; Lane 6: HCT116 Control (Untreated cells, negative control); Lane 7: PAX-treated HCT116 cells (positive control); Lane8: RJ (25 mg/mL)-treated HCT116 cells; Lane 9: EA (50 μM) -treated HCT116 cells; Lane 10: RJ (25 mg/mL) and EA (50 μM) combination-treated HCT116 cells. PAX: Paclitaxel, Scale bar: 500 μm.

Genomic DNA fragmentation of accompanied by morphological changes in the cell and nucleus is a characteristic feature of late apoptosis. During apoptosis, chromatin condensation, nuclear fragmentation, membrane blebbing, and, ultimately, the formation of apoptotic bodies can occur, and these morphological changes can be monitored throughout the process. To investigate the mechanism of action of the EA-RJ combination, a DNA laddering assay was performed on colorectal cancer cells. Based on the findings obtained from the xCELLigence RTCA MP system, 20 mg/mL of RJ and 40 μM of EA were applied separately and together for 42 h to the HT29 cell line; and 25 mg/mL of RJ and 50 μM of EA separately and together for 24 hours to the HCT116 cell line, and the cells were photographed using a Primovert inverted microscope (Zeiss, Germany). As can be seen in Figure 3, the application of RJ (Lane 8), EA (Lane 9), and the RJ-EA combination (Lane 10) to the HCT116 cell line, and the RJ-EA combination (Lane 5) to the HT29 cell line triggered apoptosis. It was thus understood that the DNA fragmentation caused by the RJ-EA combination was greater than that produced by RJ and EA alone.

### 3.4. Determination of mRNA levels of pro-apoptotic Bax and anti-apoptotic *Bcl*-2 genes

**Figure 4:**
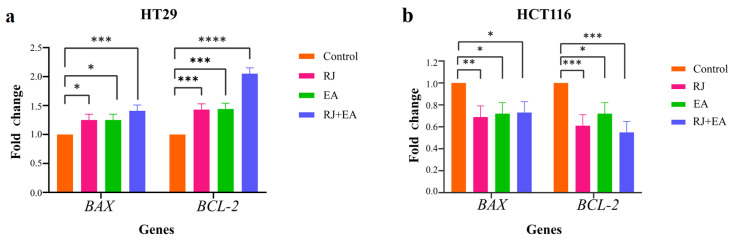
Analysis of gene expression by RT-qPCR of apoptotic pathway genes *Bax* and *Bcl-2*. HT29 cells were exposed to 20 mg/mL RJ, 40 μM EA, and 20 mgRJ+40 μM EA for 42 h (a). HCT116 cells were exposed to 25 mg/mL RJ, 50 μM EA, and 25 mg/mL RJ+50 μM EA for 24 h (b). Fold change calculations of RT-qPCR analyses were performed in Qiagen GeneGlobe (Germany). *p < 0.05, **p < 0.01, ***p < 0.001, ****p < 0.0001.

To investigate the effects of RJ, EA, and the RJ-EA combination on the expression levels of *Bax* and *Bcl-2*, the mRNA levels of *Bax* and *Bcl-2* were determined through an RT-qPCR analysis. The RT-qPCR data analysis was performed using GeneGlobe Data Analysis Software (Qiagen, Germany) to measure the relative quantification of *Bax* and *Bcl-2* expression, while GAPDH was used as an endogenous control. The results show that the RJ-EA combination significantly increased the expression of *Bax*—a proapoptotic protein—and induced the apoptotic pathway in both HT29 and HCT116 cells (Figure 4). When 25 mg/mL RJ was applied alone to HCT116 cells, the *Bax* expression was increased compared to *Bcl-2*—an antiapoptotic protein.

### 3.5. Transcriptome profiling and functional enrichment analyses of treated cells

**Figure 5:**
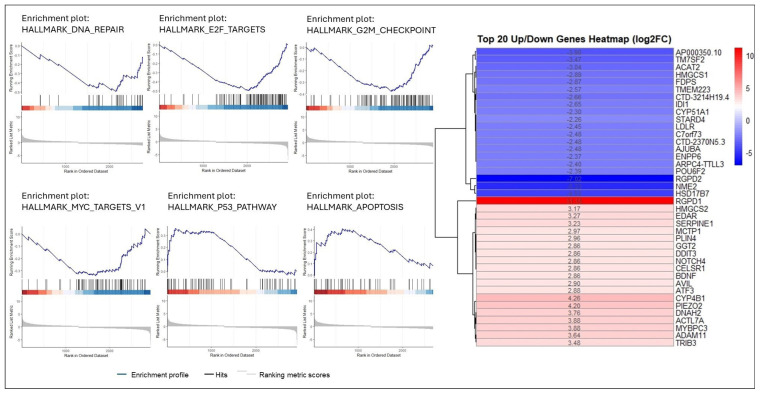
Heatmap analysis of the top 20 upregulated and downregulated genes (right) and gene set enrichment analysis (left) in HT29 cells treated with 20 mg/mL RJ and 40 μM EA for 42 h, based on RNA-seq data (|Log2FC| > 1, FDR < 0.05).

**Figure 6:**
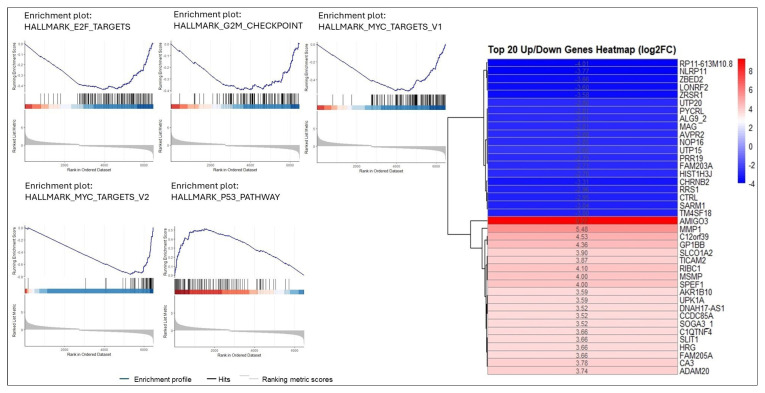
Heatmap analysis of the top 20 upregulated and downregulated genes (right) and gene set enrichment analysis (left) in HCT116 cells treated with 25 mg/mL RJ and 50 μM EA for 24 hours, based on RNA-seq data (|Log2FC| > 1, FDR < 0.05).

The total RNA-seq library results treated with the combination of 25 mg/mL RJ and 50 μM EA for 24 h for HCT116 cells, and 20 mg/mL RJ and 40 μM EA for 42 h for HT29 cells were analyzed. The results of the analysis revealed that the genes deregulated by the treatment method applied to the cell lines (|Log2FC| > 1 and FDR < 0.05) were 582 (up: 312, down: 270) in the HT29 cell lines and 1703 (up: 838, down: 865) in the HCT116 cell line (suppl.1). Heatmap analyses of the top 20 up/downregulated genes were applied to both cell lines (Figures 5 and 6)

GSEA analysis was performed on all deregulated genes (FDR < 0.05) for each cell line. In HT29 cells, hallmark apoptosis, hypoxia, and p53 pathway-related genes were most affected by EA and RJ, showing increase activation (NES > 0, p adj < 0.05). Furthermore, pathways such as hallmark DNA repair, E2F target, G2M checkpoint, oxidative phosphorylation, and MYC targets were noted to be suppressed in treated the HT29 cells (NES < 0, p adj < 0.05) (Figure 5). When the same analyses were performed on HCT116 cells, hallmark p53, hypoxia, and glycolysis mechanisms were activated, while hallmark MYC target V1–V2, E2F targets, G2M checkpoint, oxidative phosphorylation, and MYC target mechanisms were suppressed (Figure 6, suppl.1).

A gene ontology biological process analysis of genes with |Log2FC| > 1 in HT29 cells revealed that highly expressed genes following treatment were primarily involved in responses to stimuli, and endoplasmic reticulum stress, responses to organic substances, responses to chemicals, and responses to unfolded proteins. When the same analysis was performed for downregulated genes, mechanisms such as Sterol biosynthetic processes, Cholesterol biosynthetic processes, and the regulation of DNA-templated DNA replication were prominent (suppl.1). The HCT116 gene ontology biological process analysis revealed that ribosome biogenesis, ribonucleoprotein complex biogenesis, ncRNA, and RNA processing mechanisms were prominent among the downregulated genes, while mechanisms such as response to lipids, negative regulation of biological processes, and cellular responses to chemical stimulus were prominent among the upregulated genes (suppl.1).

In the KEGG pathway analyses of upregulated genes in HT29 cells to which MCODE PPI analyses were applied, protein processing in the endoplasmic reticulum, the pathways implicated also in neurological disorders, and apoptosis were identified, whereas in the downregulated genes, pathways such as steroid biosynthesis, the metabolic pathways in MCODE cluster 1, DNA replication, oxidative phosphorylation, and thermogenesis in cluster 2 were prominent (Table 2). Upregulated genes were shown to be particularly active in animal mitophagy, protein digestion and absorption, and in the IL-17 signaling pathway in the analysis of HCT116. The analysis of downregulated genes revealed such important processes as RNA polymerase and ribosome synthesis in eukaryotes (Table 3).

**Figure 7:**
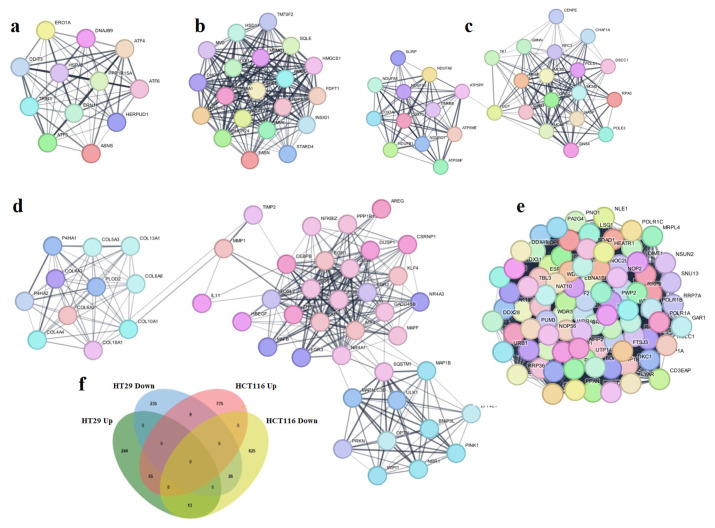
PPI images for HT29 and HCT116, respectively **a:** (up-regulated genes in HT29) **b–c:** (down-regulated genes in HT29), **d:** (up-regulated genes in HCT116), **e:** (down-regulated genes in HCT116) and, **f:** Venn diagram analysis of HT29 and HCT116 treated cells.

To better understand the combined effect of EA and RJ treatments on gene expression, a Venn diagram analysis was performed for the identification of DEGs in the HT29 and HCT116 cell lines. These analyses revealed 55 upregulated genes and 26 downregulated genes in both cell lines (Figure 7). When the mechanisms in which these genes are involved were investigated using the STRING tool, it was determined that the activated genes direct the ferroptosis mechanism *(GCLC*, *SLC7A11*, *TFRC*, *FTH1)* and intrinsic apoptotic pathway *(ERO1A*, *CEBPB*, *DDIT3*, *DDIT4*, *TRIB3*, *PPP1*, *ERN1*, *CHAC1)* while the downregulated genes are involved in DNA replication and cell cycle mechanisms (*POLE3*, *MCM2*, *MCM3*, *MCM5*, *MCM6*).

## 4. Discussion

Despite the advances achieved in recent years; cancer continues to be a global health problem with high morbidity and mortality rates. Colorectal cancer is the third most common form of cancer worldwide after lung and breast cancer (Xi and Xu 2021). The chemotherapeutics currently in use for the treatment of colorectal cancer have significant side effects, aside from their high toxicity, and response rates to these drugs are gradually decreasing (Lin et al., 2020; Wang et al., 2022). Recent efforts in the fight against cancer have focused on the research and development of new chemotherapeutics that can kill cancer cells with high treatment potential, but with low toxic effects on healthy cells (Tanaka et al.,2023). In the present study, the anticancer potential and mechanisms of action of two natural products of plant and animal origin with high antioxidant capacity, used in combination, were investigated. Widely available natural products play an extremely important role in the development of new anticancer agents, and have been identified with multiple pharmacological activities, rich active ingredients, and very few side effects. Natural products obtained from such organisms such as plants, fungi, animals and bacteria are today used in the treatment of many diseases, including cancer. For example, anticancer drugs used such as paclitaxel, irinotecan, etoposide, and vincristine are all obtained from natural sources (Calixto 2019; Patra et al., 2021). In the present study, the treatment effects of RJ and EA were studied, individually and in combination, and their effectiveness against the BEAS-2B, HCT116 and HT29 cell lines was evaluated. RJ, which is sourced from honeybees, contains such bioactive compounds as 10-hydroxy-2-decenoic acid (10-HDA), and has been shown to have potential anticancer properties and synergistic effects when used with chemotherapeutic agents (Albalawi et al., 2021; Salama et al., 2022). Although both HT29 and HCT116 are colorectal cancer cell lines, HT29 is the more aggressive of the two, being a metastatic cell line with a different structure to HCT116. EA and RJ demonstrated significant antiproliferative activity in HCT116 cells after 24 hours, and after 42 h in HT29 cells, supporting the well-known broad-spectrum anticancer activities of EA, including its anti-proliferative, pro-apoptotic, anti-angiogenic, and anti-metastatic effects (Ceci et al., 2018; Ríos et al., 2018; Čižmáriková et al., 2023). While the two agents reduced proliferation in non-tumorigenic BEAS-2B cells, their cytotoxic effects were limited. The combined application of RJ-EA to HT29 and HCT116 cell lines induced greater antiproliferative activity and reduced the cell index more than their use individually (Figure1). Previous studies have reported that EA increases anticancer activity in the HeLa cell line when used with curcumin, which is less bioavailable (Kumar et al., 2016), and similar synergistic effects have been observed with RJ combined with such other agents as thymoquinone in breast cancer (Moubarak et al., 2021). The epigenetic changes that occur during carcinogenesis can be reversed by phytochemicals. Furthermore, combinations of phytochemicals can suppress cell proliferation and metastasis, and can lead the cell to apoptosis (Patra et al., 2021; Islam et al., 2022; Asma et al., 2022; Tanaka et al., 2023). In most cancers, changes in cell metabolism occur to support the increased proliferation, and one of the mechanisms underlying antiproliferative activity may be related to the disrupted energy metabolism. Aerobic glycolysis is one of the main features of the tumor cell metabolism in colorectal cancer, which can be attributed to the fact that cancer cells strive to maintain low oxidative stress by avoiding oxidative phosphorylation, which leads to the formation of more free oxygen radicals (Di Gregorio et al., 2022). ECAR is a measure of the lactic acid levels that result from the conversion of glucose to lactate during glycolysis, and can highlight the presence of glycolytic inhibition. In the present study, EA and RJ were applied separately to each cell line, and produced similar ECAR results. When used in combination, however, the two agents significantly reduced the ECAR rate, especially in HCT116, effectively inhibiting aerobic glycolysis (Figure 2). Previous studies in the literature have reported that Galloflavin applied in combination with EA regulates the glycolytic pathway by increasing the expression of SIRT6, resulting in an anticancer effect (Rahnasto-Rilla et al., 2020). EA has also been reported to modulate other key metabolic pathways, including AMPK/mTOR, which are crucial in cancer cell survival and proliferation (Ni et al., 2023). For cancer cells with mitochondrial defects and those in hypoxic environments, glycolytic inhibitors are of particular interest as a new class of anticancer agent. An examination of DNA laddering and cell images revealed that the RJ-EA combination induced apoptosis in HCT116, to a significant degree (Figure 3). The RJ-EA combination would appear to have an apoptotic effect on HT29 cells, albeit to a lesser extent than HCT116, and this is also evidenced by the expression of pro-apoptotic *Bax* and anti-apoptotic *Bcl-2* genes. The RJ-EA combination was also noted to increase the *Bax*/*Bcl-2* ratio in a comparison with in which EA or RJ were applied individually. While *Bcl-2* expression exceeded that of *Bax* only in the EA and RJ treatments of HT29 cells, the EA-RJ combination induced the apoptotic pathway in HCT116 cells by increasing the *Bax*/*Bcl-2* ratio (Figure 4). GSEA, PPI, Gene Ontology and KEGG pathway analyses were performed to examine the general transcriptome profile associated with the synergistic effects of EA and RJ on both cell lines. In the GSEA analyses, the increase in apoptosis and p53 pathway-related genes and the suppression of genes involved in E2F target, G2M checkpoint, oxidative phosphorylation, MYC target mechanism with the treatment applied in both cell lines suggested a directly proportional relationship with the antiproliferative effect on cancer cells and increased apoptosis. The combination of these two treatments is believed to prevent colon cancer cells from proliferating and to have an effect that triggers apoptosis through their ability to inhibit the cell cycle or to reduce the efficacy of the regulatory points of the cell cycle. Furthermore, the downregulation of most genes involved in DNA repair in HT29 cells points to an accumulation of cell defects, leading to apoptotic induction. The suppression of genes in the MYC target mechanism and their interactions with other mechanisms have been found to have a detrimental effect on cancer cells, despite the activation of the genes involved in the MTORC1 mechanism, which is important for cell growth and division. An analysis of the 20 most common downregulated genes in HT29 cells revealed several that are involved in cholesterol metabolic processes (HMGCS1, TM7SF2, CYP51A1, FDPS, IDI1, HSD17B7, LDLR). Clinical and experimental studies have shown that changes in the cholesterol metabolism play a role in cancer development, and that increased cholesterol levels are associated with higher incidences of cancer. Furthermore, cholesterol-lowering drugs (e.g. statins) have been shown to reduce risk and lower the mortality associated with cancers, particularly in the breast, prostate, and colorectal region (Silvente-Poirot et al., 2012; Ding et al., 2019). It has also been reported that the activation of the cholesterol metabolism activates oncogenic signaling pathways (Ding et al., 2019). Xu et al., (2021) reported that the overexpression of TM7SF2 inhibited cell apoptosis, promoted cell proliferation and metastasis, and avoided G0/G1 phase arrest in C33A and SiHa cells (Xu et al., 2021). However, when TM7SF2 was knocked down, the opposite effects were seen in cervical cancer cells. In a pan-cancer study, HMGCS1 mRNA expression was noted to be increased in most digestive tract-related cancers, including colon adenocarcinoma (COAD), esophageal carcinoma (ESCA), and rectal adenocarcinoma (READ) (Zhou et al., 2022). Zhou et al. further reported HMGCS1 to be overexpressed in colon cancer cells and tissues, and to promote proliferation, migration, and invasion (Zhou et al., 2020). The therapeutic targeting of HMGCS1 may improve MEK inhibitor sensitivity and overcome the drug resistance mediated by RAF/RAS mutations, which may be a promising therapeutic strategy for colon cancer patients. All of the above studies suggest that the suppression of genes involved in the cholesterol metabolism have a negative effect on tumor growth, proliferation, and prognosis. Atop 20 up-down gene analysis of HCT116 cells revealed that downregulated genes in particular (UTP15, UTP20, RRS1, NOP16) play a role in ribosome biogenesis. According to a study by Wu et al. (2017), RRS1 expression is considerably higher in CRC tissues than in nearby normal tissues, and is associated with a lower overall survival rate in CRC patients (Wu et al., 2017). The critical role of RRS1 in colorectal cancer (CRC) progression has been highlighted in studies showing that its knockdown inhibits growth, leads to G2/M arrest, and induces apoptosis in such CRC cell lines as HCT116 and RKO (Wu et al., 2017). RRS1 reduction inhibited the growth of RKO and HCT116 CRC cells, leading to G2/M cell cycle arrest and death (Wu et al., 2017). Another study reported that RRS1 silencing significantly reduced cell proliferation, inhibited the cell cycle, and promoted apoptosis in a papillary thyroid carcinoma cell line. Furthermore, the silencing of RRS1 upregulated the genes related to apoptosis and metabolism, and repressed the genes related to cell proliferation and blood vessel development (Chen et al., 2018). A Venn diagram analysis revealed an increased presence of genes associated with the apoptotic process in both cell lines following treatment, suggesting a significant and positive contribution of EA and RJ to the apoptotic process (Figure 7). In addition, the suppression of the cell cycle and DNA replication systems in commonly downregulated genes (*MCMs*) indicates that the combined effect of these two molecules can contribute significantly to cancer treatments. There is a need, however, for in vivo studies into their synergistic effects (Shakib Khoob et al., 2022).

## Supplementary Information



## Figures and Tables

**Table 1:** Oligonucleotide primer sets for RT-qPCR.

Gene	Accession no.	Forward sequence	Reverse sequence	T_M_
GAPDH	NM_002046.5	GTCTCCTCTGACTTCAACAGCG	ACCACCCTGTTGCTGTAGCCAA	60°C
*BAX*	NM_004324.3	TCTGACGGCAACTTCAACTG	TTGAGGAGTCTCCCCAACC	60°C
*BCL-2*	NM_000633.2	ATCGCCCTGTGGATGACTGAGT	GCCAGGAGAAATCAAACAGAGGC	60°C

**Table 2:** PPI and KEGG pathway analyses of HT29 up/downregulated genes via MCODE.

Upregulated genes (Cluster 1 – score: 11,455)					
Kegg pathway	Description	FDR value	Genes	Background genes	Gene names
hsa04141	Protein processing in endoplasmic reticulum	4.39E-12	8	163	*[PPP1R15A*, *HSPA5*, *ATF4*, *ATF6*, *ERO1A*, *ERN1*, *HERPUD1*, *DDIT3]*
hsa05012	Parkinson disease	3.25E-5	5	236	*[HSPA5*, *ATF4*, *ATF6*, *ERN1*, *DDIT3]*
hsa05014	Amyotrophic lateral sclerosis	1.5E-4	5	350	*[HSPA5*, *ATF4*, *ATF6*, *ERN1*, *DDIT3]*
hsa05010	Alzheimer disease	0.004	4	354	*[ATF4*, *ATF6*, *ERN1*, *DDIT3]*
hsa04210	Apoptosis	0.0043	3	131	*[ATF4*, *ERN1*, *DDIT3]*
hsa04932	Nonalcoholic fatty liver disease	0.005	3	146	*[ATF4*, *ERN1*, *DDIT3]*
hsa05020	Prion disease	0.0234	3	263	*[HSPA5*, *ATF4*, *DDIT3]*
hsa04918	Thyroid hormone synthesis	0.0386	2	73	*[HSPA5*, *ATF4]*
**Downregulated genes (Cluster 1 – score: 19,263)**					
hsa00100	Steroid biosynthesis	4.51E-19	9	20	*[CYP51A1*, *HSD17B7*, *MSMO1*, *SQLE*, *TM7SF2*, *DHCR7*, *NSDHL*, *DHCR24*, *FDFT1]*
hsa01100	Metabolic pathways	7.8E-15	17	1435	*[CYP51A1*, *MVK*, *HSD17B7*, *MSMO1*, *SQLE*, *TM7SF2*, *HMGCR*, *MVD*, *FASN*, *HMGCS1*, *DHCR7*, *FDPS*, *ACAT2*, *NSDHL*, *DHCR24*, *IDI1*, *FDFT1]*
hsa00900	Terpenoid backbone biosynthesis	5.82E-14	7	22	*[MVK*, *HMGCR*, *MVD*, *HMGCS1*, *FDPS*, *ACAT2*, *IDI1]*
hsa00072	Synthesis and degradation of ketone bodies	0.0054	2	10	*[HMGCS1*, *ACAT2]*
hsa00650	Butanoate metabolism	0.0262	2	27	*[HMGCS1*, *ACAT2]*
**Downegulated genes (Cluster 2 – score: 12,621)**					
hsa03030	DNA replication	5.92E-13	8	36	*[MCM5*, *RPA3*, *MCM6*, *MCM2*, *POLE3*, *RFC3*, *POLD1*, *MCM3]*
hsa00190	Oxidative phosphorylation	9.02E-13	10	128	*[NDUFS6*, *ATP5MF*, *ATP5ME*, *COX7A2*, *NDUFS5*, *NDUFA8*, *ATP5PF*, *COX7C*, *NDUFC1*, *NDUFB1]*
hsa04714	Thermogenesis	1.35E-10	10	226	*[NDUFS6*, *ATP5MF*, *ATP5ME*, *COX7A2*, *NDUFS5*, *NDUFA8*, *ATP5PF*, *COX7C*, *NDUFC1*, *NDUFB1]*
hsa04932	Nonalcoholic fatty liver disease	1.9E-7	7	146	*[NDUFS6*, *COX7A2*, *NDUFS5*, *NDUFA8*, *COX7C*, *NDUFC1*, *NDUFB1]*
hsa05012	Parkinson disease	1.9E-7	8	236	*[NDUFS6*, *COX7A2*, *NDUFS5*, *NDUFA8*, *ATP5PF*, *COX7C*, *NDUFC1*, *NDUFB1]*
hsa05020	Prion disease	2.88E-7	8	263	*[NDUFS6*, *COX7A2*, *NDUFS5*, *NDUFA8*, *ATP5PF*, *COX7C*, *NDUFC1*, *NDUFB1]*
hsa05016	Huntington disease	5.91E-7	8	295	*[NDUFS6*, *COX7A2*, *NDUFS5*, *NDUFA8*, *ATP5PF*, *COX7C*, *NDUFC1*, *NDUFB1]*
hsa05010	Alzheimer disease	1.89E-6	8	354	*[NDUFS6*, *COX7A2*, *NDUFS5*, *NDUFA8*, *ATP5PF*, *COX7C*, *NDUFC1*, *NDUFB1]*
hsa05014	Amyotrophic lateral sclerosis	1.89E-6	8	350	*[NDUFS6*, *COX7A2*, *NDUFS5*, *NDUFA8*, *ATP5PF*, *COX7C*, *NDUFC1*, *NDUFB1]*
hsa03420	Nucleotide excision repair	3.2E-5	4	46	*[RPA3*, *POLE3*, *RFC3*, *POLD1]*

**Table 3:** PPI and KEGG pathway analyses of HCT116 up/downregulated genes via MCODE.

Upregulated genes (Cluster 1 – score: 12,766)					
Kegg pathway	Description	FDR value	Genes	Background genes	Gene names
hsa04137	Mitophagy - animal	2.36E-9	8	64	*[ULK1*, *NBR1*, *PRKN*, *JUN*, *PINK1*, *OPTN*, *BNIP3L*, *SQSTM1]*
hsa04974	Protein digestion and absorption	3.25E-8	8	100	*[COL5A3*, *COL6A3*, *COL10A1*, *COL6A6*, *COL18A1*, *COL4A3*, *COL4A4*, *COL13A1]*
hsa04380	Osteoclast differentiation	6.61E-5	6	120	*[FOSB*, *FOSL2*, *JUNB*, *FOS*, *JUN*, *SQSTM1]*
hsa04657	IL-17 signaling pathway	3.0E-4	5	91	*[FOSB*, *CEBPB*, *FOS*, *MMP1*, *JUN]*
hsa04926	Relaxin signaling pathway	0.0011	5	126	*[FOS*, *MMP1*, *JUN*, *COL4A3*, *COL4A4]*
hsa04010	MAPK signaling pathway	0.0032	6	286	*[GADD45B*, *DUSP1*, *FOS*, *JUN*, *AREG*, *NR4A1]*
hsa04512	ECM-receptor interaction	0.0032	4	88	*[COL6A3*, *COL6A6*, *COL4A3*, *COL4A4]*
hsa05210	Colorectal cancer	0.0032	4	82	*[GADD45B*, *FOS*, *JUN*, *AREG]*
hsa05323	Rheumatoid arthritis	0.0032	4	83	*[IL11*, *FOS*, *MMP1*, *JUN]*
hsa04933	AGE-RAGE signaling pathway in diabetic complications	0.0034	4	96	*[EGR1*, *JUN*, *COL4A3*, *COL4A4]*
**Downregulated genes (Cluster 1 – score: 80,276)**					
hsa03008	Ribosome biogenesis in eukaryotes	5.31E-37	26	77	*[UTP18*, *GAR1*, *MPHOSPH10*, *NAT10*, *IMP4*, *NOP58*, *LSG1*, *NHP2*, *PWP2*, *UTP15*, *NOL6*, *WDR3*, *WDR75*, *RRP7A*, *UTP4*, *GTPBP4*, *HEATR1*, *DKC1*, *NOP56*, *UTP14A*, *SNU13*, *WDR43*, *GNL3*, *WDR36*, *TBL3*, *EMG1]*
hsa03020	RNA polymerase	0.0029	4	31	*[TWISTNB*, *POLR1A*, *POLR1B*, *POLR1C]*

## Data Availability

The datasets generated during and/or analyzed during the current study are available from the corresponding author on reasonable request.

## References

[b1-tjb-49-06-675] Albalawi AE Althobaiti NA Alrdahe SS Alhasani RH Alaryani FS 2021 Anti-Tumor Effects of Queen Bee Acid (10-Hydroxy-2-Decenoic Acid) Alone and in Combination with Cyclophosphamide and Its Cellular Mechanisms against Ehrlich Solid Tumor in Mice Molecules 26 22 7021 10.3390/molecules26227021 34834112 PMC8617861

[b2-tjb-49-06-675] Alhosin M (2023) Epigenetics Mechanisms of Honeybees: Secrets of Royal Jelly Epigenetics Insights 16 25168657231213717 10.1177/25168657231213717 38033464 PMC10687967

[b3-tjb-49-06-675] Asma ST Acaroz U Imre K Morar A Shah SRA 2022 Natural Products/Bioactive Compounds as a Source of Anticancer Drugs Cancers (Basel) 14 24 6203 10.3390/cancers14246203 36551687 PMC9777303

[b4-tjb-49-06-675] Baptista BG Lima LS Ribeiro M Britto IK Alvarenga L (2023) Royal jelly: a predictive, preventive and personalised strategy for novel treatment options in non-communicable diseases EPMA Journal 14 3 381 404 10.1007/s13167-023-00330-8 37605655 PMC10439876

[b5-tjb-49-06-675] Borawska MH Markiewicz-Żukowska R Naliwajko SK Moskwa J Bartosiuk E 2014 The interaction of bee products with temozolomide in human diffuse astrocytoma, glioblastoma multiforme and astroglia cell lines Nutrition and Cancer 66 7 1247 56 10.1080/01635581.2014.951735 25256634

[b6-tjb-49-06-675] Calixto JB 2019 The role of natural products in modern drug discovery Anais da Academia Brasileira de Ciências 91 Suppl 3 e20190105 10.1590/0001-3765201920190105 31166478

[b7-tjb-49-06-675] Ceci C Lacal PM Tentori L De Martino MG Miano R 2018 Experimental Evidence of the Antitumor, Antimetastatic and Antiangiogenic Activity of Ellagic Acid Nutrients 10 11 1756 10.3390/nu10111756 30441769 PMC6266224

[b8-tjb-49-06-675] Chakrabarti S Peterson CY Sriram D Mahipal A (2020) Early stage colon cancer: Current treatment standards, evolving paradigms, and future directions World Journal of Gastrointestinal Oncology 12 8 808 832 10.4251/wjgo.v12.i8.808 32879661 PMC7443846

[b9-tjb-49-06-675] Chen P Chen F Zhou B 2019 Antioxidative, anti-inflammatory and anti-apoptotic effects of ellagic acid in liver and brain of rats treated by D-galactose Scientific Reports 8 1 1465 10.1038/s41598-018-19732-0 PMC5780521 29362375

[b10-tjb-49-06-675] Chen F Jin Y Feng L Zhang J Tai J RRS1 gene expression involved in the progression of papillary thyroid carcinoma 2018 Cancer Cell International 18 20 10.1186/s12935-018-0519-x 29449788 PMC5812111

[b11-tjb-49-06-675] Čižmáriková M Michalková R Mirossay L Mojžišová G Zigová M 2023 Ellagic Acid and Cancer Hallmarks: Insights from Experimental Evidence Biomolecules 13 11 1653 10.3390/biom13111653 38002335 PMC10669545

[b12-tjb-49-06-675] Deepika Maurya PK 2022 Ellagic acid: insight into its protective effects in age-associated disorders 3 Biotech 12 12 340 10.1007/s13205-022-03409-7 PMC9633905 36340805

[b13-tjb-49-06-675] Di Gregorio J Petricca S Iorio R Toniato E Flati V 2022 Mitochondrial and metabolic alterations in cancer cells European Journal of Cell Biology 101 3 151225 10.1016/j.ejcb.2022.151225 35453093

[b14-tjb-49-06-675] Ding X Zhang W Li S Yang H 2019 The role of cholesterol metabolism in cancer American Journal of Cancer Research 2 219 227 30906624 PMC6405981

[b15-tjb-49-06-675] Evtyugin DD Magina S Evtuguin DV 2020 Recent Advances in the Production and Applications of Ellagic Acid and Its Derivatives A Review Molecules 25 12 2745 10.3390/molecules25122745 PMC7355634 32545813

[b16-tjb-49-06-675] Gil TY Hong CH An HJ 2021 Anti-Inflammatory Effects of Ellagic Acid on Keratinocytes via MAPK and STAT Pathways International Journal of Molecular Sciences 22 3 1277 10.3390/ijms22031277 33525403 PMC7865693

[b17-tjb-49-06-675] Islam MR Akash S Rahman MM Nowrin FT Akter T (2022) Colon cancer and colorectal cancer: Prevention and treatment by potential natural products Chemico-biological Interactions 368 110170 10.1016/j.cbi.2022.110170 36202214

[b18-tjb-49-06-675] Kaur H Ghosh S Kumar P Basu B Nagpal K (2021) Ellagic acid-loaded, tween 80-coated, chitosan nanoparticles as a promising therapeutic approach against breast cancer: In-vitro and in-vivo study Life Sciences 284 119927 10.1016/j.lfs.2021.119927 34492262

[b19-tjb-49-06-675] Kumar D Basu S Parija L Rout D Manna 2016 Curcumin and Ellagic acid synergistically induce ROS generation, DNA damage, p53 accumulation and apoptosis in HeLa cervical carcinoma cells Biomedicine & Pharmacotherapy 81 31 37 10.1016/j.biopha.2016.03.037 27261574

[b20-tjb-49-06-675] Leowattana W Leowattana P Leowattana T (2023) Systemic treatment for metastatic colorectal cancer World Journal of Gastroenterology 29 10 1569 1588 10.3748/wjg.v29.i10.1569 36970592 PMC10037252

[b21-tjb-49-06-675] Li LW Na C Tian SY Chen J Ma R 2018 Ellagic acid induces HeLa cell apoptosis via regulating signal transducer and activator of transcription 3 signaling Experimental and Therapeutic Medicine 16 1 29 36 10.3892/etm.2018.6182 29896225 PMC5995030

[b22-tjb-49-06-675] Lin SR Chang CH Hsu CF Tsai MJ Cheng H (2020) Natural compounds as potential adjuvants to cancer therapy: Preclinical evidence British Journal of Pharmacology 177 6 1409 1423 10.1111/bph.14816 31368509 PMC7056458

[b23-tjb-49-06-675] Lu G Wang X Cheng M Wang S Ma K (2023) The multifaceted mechanisms of ellagic acid in the treatment of tumors: State-of-the-art Biomedicine & Pharmacotherapy 165 115132 10.1016/j.biopha.2023.115132 37423169

[b24-tjb-49-06-675] Maleki V Abbaszadeh S Seyyed Shoura SM Sohrabnavi A Sepandi M (2023) Potential roles of ellagic acid on metabolic variables in diabetes mellitus: A systematic review Clinical and Experimental Pharmacology & Physiology 50 2 121 131 10.1111/1440-1681.13729 36222179

[b25-tjb-49-06-675] Mannino F Imbesi C Bitto A Minutoli L Squadrito F (2023) Anti-oxidant and anti-inflammatory effects of ellagic and punicic acid in an in vitro model of cardiac fibrosis Biomedicine & Pharmacotherapy 162 114666 10.1016/j.biopha.2023.114666 37030134

[b26-tjb-49-06-675] Mansouri Z Dianat M Radan M Badavi M (2020) Ellagic Acid Ameliorates Lung Inflammation and Heart Oxidative Stress in Elastase-Induced Emphysema Model in Rat Inflammation 43 3 1143 1156 10.1007/s10753-020-01201-4 32103438

[b27-tjb-49-06-675] Mansourizadeh F Alberti D Bitonto V Tripepi M Sepehri H 2020 Efficient synergistic combination effect of Quercetin with Curcumin on breast cancer cell apoptosis through their loading into Apo ferritin cavity Colloids and Surfaces. B, Biointerfaces 191 110982 10.1016/j.colsurfb.2020.110982 32220813

[b28-tjb-49-06-675] Mohammadinejad A Mohajeri T Aleyaghoob G Heidarian F Kazemi Oskuee R (2022) Ellagic acid as a potent anticancer drug: A comprehensive review on in vitro, in vivo, in silico, and drug delivery studies Biotechnology and Applied Biochemistry 69 6 2323 2356 10.1002/bab.2288 34846078

[b29-tjb-49-06-675] Morgan E Arnold M Gini A Lorenzoni V Cabasag CJ (2023) Global burden of colorectal cancer in 2020 and 2040: incidence and mortality estimates from GLOBOCAN Gut 72 2 338 344 10.1136/gutjnl-2022-327736 36604116

[b30-tjb-49-06-675] Moubarak MM Chanouha N Abou Ibrahim N Khalife H Gali-Muhtasib H (2021) Thymoquinone anticancer activity is enhanced when combined with royal jelly in human breast cancer World Journal of Clinical Oncology 12 5 342 354 10.5306/wjco.v12.i5.342 34131566 PMC8173327

[b31-tjb-49-06-675] Muthukumaran S Tranchant C Shi J Ye X Xue SJ 2017 Ellagic acid in strawberry (Fragaria spp.): Biological, technological, stability, and human health aspects Food Quality and Safety 1 4 227 252 10.1093/fqsafe/fyx023

[b32-tjb-49-06-675] Naghibi N Sadeghi A Movahedinia S Rahimi Naiini M Rajizadeh MA (2023) Ellagic acid ameliorates aging-induced renal oxidative damage through upregulating SIRT1 and NRF2 BMC Complementary Medicine and Therapies 23 1 77 10.1186/s12906-023-03907-y 36899375 PMC9999491

[b33-tjb-49-06-675] Ni X Shang FS Wang TF Wu DJ Chen DG (2023) Ellagic acid induces apoptosis and autophagy in colon cancer through the AMPK/mTOR pathway Tissue Cell 81 102032 10.1016/j.tice.2023.102032 36701898

[b34-tjb-49-06-675] Park SW Kwon MJ Yoo JY Choi HJ Ahn YJ 2014 Antiviral activity and possible mode of action of ellagic acid identified in Lagerstroemia speciosa leaves toward human rhinoviruses BMC Complementary and Alternative Medicine 14 171 10.1186/1472-6882-14-171 24885569 PMC4052798

[b35-tjb-49-06-675] Patra S Pradhan B Nayak R Behera C Das S (2021) Dietary polyphenols in chemoprevention and synergistic effect in cancer: Clinical evidences and molecular mechanisms of action Phytomedicine 90 153554 10.1016/j.phymed.2021.153554 34371479

[b36-tjb-49-06-675] Rahnasto-Rilla M Järvenpää J Huovinen M Schroderus AM Ihantola EL (2020) Effects of galloflavin and ellagic acid on sirtuin 6 and its anti-tumorigenic activities Biomedicine & Pharmacotherapy 131 110701 10.1016/j.biopha.2020.110701 32905943 PMC12036747

[b37-tjb-49-06-675] Ríos JL Giner RM Marín M Recio MC 2018 A Pharmacological Update of Ellagic Acid Planta Medica 84 15 1068 1093 10.1055/a-0633-9492 29847844

[b38-tjb-49-06-675] Salama S Shou Q Abd El-Wahed AA Elias N Xiao J 2022 Royal Jelly: Beneficial Properties and Synergistic Effects with Chemotherapeutic Drugs with Particular Emphasis in Anticancer Strategies Nutrients 14 19 4166 10.3390/nu14194166 36235818 PMC9573021

[b39-tjb-49-06-675] Savic IM Jocic E Nikolic VD Popsavin MM Rakic SJ 2019 The effect of complexation with cyclodextrins on the antioxidant and antimicrobial activity of ellagic acid Pharmaceutical Development and Technology 24 4 410 418 10.1080/10837450.2018.1502318 30035651

[b40-tjb-49-06-675] Shakib Khoob M Hosseini SM Kazemi S (2022) In Vitro and In Vivo Antioxidant and Anticancer Potentials of Royal Jelly for Dimethylhydrazine-Induced Colorectal Cancer in Wistar Rats Oxidative Medicine and Cellular Longevity 2022 9506026 10.1155/2022/9506026 35910834 PMC9334054

[b41-tjb-49-06-675] Silvente-Poirot S Poirot M 2012 Cholesterol metabolism and cancer: the good, the bad and the ugly Current Opinion in Pharmacology 12 673 676 10.1016/j.coph.2012.10.004 23103112

[b42-tjb-49-06-675] Tanaka T Aoki R Terasaki M 2023 Potential Chemopreventive Effects of Dietary Combination of Phytochemicals against Cancer Development Pharmaceuticals (Basel) 16 11 1591 10.3390/ph16111591 38004456 PMC10674766

[b43-tjb-49-06-675] Thompson HJ Lutsiv T (2023) Natural Products in Precision Oncology: Plant- Based Small Molecule Inhibitors of Protein Kinases for Cancer Chemoprevention Nutrients 15 5 1192 10.3390/nu15051192 36904191 PMC10005680

[b44-tjb-49-06-675] Tošović J Bren U 2020 Antioxidative Action of Ellagic Acid-A Kinetic DFT Study Antioxidants (Basel) 9 7 587 10.3390/antiox9070587 32640518 PMC7402119

[b45-tjb-49-06-675] Wang Q Shen X Chen G Du J 2022 Drug Resistance in Colorectal Cancer: From Mechanism to Clinic Cancers (Basel) 14 12 2928 10.3390/cancers14122928 35740594 PMC9221177

[b46-tjb-49-06-675] Wu XL Yang ZW He L Dong PD Hou MX 2017 RRS1 silencing suppresses colorectal cancer cell proliferation and tumorigenesis by inhibiting G2/M progression and angiogenesis Oncotarget 15 8 47 82968 82980 10.18632/oncotarget.20897 29137316 PMC5669942

[b47-tjb-49-06-675] Xi Y Xu P (2021) Global colorectal cancer burden in 2020 and projections to 2040 Translational Oncology 14 10 101174 10.1016/j.tranon.2021.101174 34243011 PMC8273208

[b48-tjb-49-06-675] Xu Y Chen X Pan S Wang ZW Zu X (2021) TM7SF2 regulates cell proliferation and apoptosis by activation of C-Raf/ERK pathway in cervical cancer Cell Death Discovery 7 299 10.1038/s41420-021-00689-5 34667152 PMC8526692

[b49-tjb-49-06-675] Zahin M Ahmad I Gupta RC Aqil F 2014 Punicalagin and ellagic acid demonstrate antimutagenic activity and inhibition of benzo[a]pyrene induced DNA adducts BioMed Research International 467465 10.1155/2014/467465 24949451 PMC4052943

[b50-tjb-49-06-675] Zhang S Shao Q Geng H Su S 2017 The effect of royal jelly on the growth of breast cancer in mice Oncology Letters 14 6 7615 7621 10.3892/ol.2017.7078 29344209 PMC5755229

[b51-tjb-49-06-675] Zhou C Wang Z Cao Y Zhao L 2022 Pan-cancer analysis reveals the oncogenic role of 3-hydroxy-3-methylglutaryl-CoA synthase 1 Cancer Reports 5 9 e1562 10.1002/cnr2.1562 34549901 PMC9458500

[b52-tjb-49-06-675] Zhou S Xu H Tang Q Xia H Bi F (2020) Dipyridamole enhances the Cytotoxicities of Trametinib against Colon Cancer cells through combined targeting of HMGCS1 and MEK pathway Molecular Cancer Therapeutics 19 1 135 146 10.1158/1535-7163.MCT-19-0413 31554653

